# Prediction of Cross-Recognition of Peptide-HLA A2 by Melan-A-Specific Cytotoxic T Lymphocytes Using Three-Dimensional Quantitative Structure-Activity Relationships

**DOI:** 10.1371/journal.pone.0065590

**Published:** 2013-07-16

**Authors:** Theres Fagerberg, Vincent Zoete, Sebastien Viatte, Petra Baumgaertner, Pedro M. Alves, Pedro Romero, Daniel E. Speiser, Olivier Michielin

**Affiliations:** 1 Department of Oncology and Ludwig Center for Cancer Research, University of Lausanne, Lausanne, Switzerland; 2 Swiss Institute of Bioinformatics, Quartier Sorge – Bâtiment Génopode, Lausanne, Switzerland; 3 National Center of Competence in Research (NCCR) Molecular Oncology, Epalinges, Switzerland; 4 Multidisciplinary Oncology Center, Lausanne University Hospital (CHUV), Lausanne, Switzerland; University of Edinburgh, United Kingdom

## Abstract

The cross-recognition of peptides by cytotoxic T lymphocytes is a key element in immunology and in particular in peptide based immunotherapy. Here we develop three-dimensional (3D) quantitative structure-activity relationships (QSARs) to predict cross-recognition by Melan-A-specific cytotoxic T lymphocytes of peptides bound to HLA A*0201 (hereafter referred to as HLA A2). First, we predict the structure of a set of self- and pathogen-derived peptides bound to HLA A2 using a previously developed *ab initio* structure prediction approach [Fagerberg *et al.*, J. Mol. Biol., 521–46 (2006)]. Second, shape and electrostatic energy calculations are performed on a 3D grid to produce similarity matrices which are combined with a genetic neural network method [So *et al.*, J. Med. Chem., 4347–59 (1997)] to generate 3D-QSAR models. The models are extensively validated using several different approaches. During the model generation, the leave-one-out cross-validated correlation coefficient (*q* ^2^) is used as the fitness criterion and all obtained models are evaluated based on their *q* ^2^ values. Moreover, the best model obtained for a partitioned data set is evaluated by its correlation coefficient (*r* = 0.92 for the external test set). The physical relevance of all models is tested using a functional dependence analysis and the robustness of the models obtained for the entire data set is confirmed using y-randomization. Finally, the validated models are tested for their utility in the setting of rational peptide design: their ability to discriminate between peptides that only contain side chain substitutions in a single secondary anchor position is evaluated. In addition, the predicted cross-recognition of the mono-substituted peptides is confirmed experimentally in chromium-release assays. These results underline the utility of 3D-QSARs in peptide mimetic design and suggest that the properties of the unbound epitope are sufficient to capture most of the information to determine the cross-recognition.

## Introduction

Antigenic peptides bound to Major Histocompatibility Complex (MHC) class I molecules on the surface of antigen presenting cells are recognized by the αβ T cell receptor (TCR) on cytotoxic T lymphocytes (CTLs) and induce the specific CD8^+^ T cell immune response against virus infected cells and tumor cells.

The αβ TCRs recognize the peptide-MHC class I complexes with a certain degree of specificity that is determined by the peptide amino acid sequence and the MHC class I allele; it has been shown that the comprehensive response to foreign antigens requires some level of cross-recognition, or cross-reactivity, such that one TCR can recognize a number of different peptides in a same MHC [1]. Interestingly, sequence homology is not necessarily needed for cross-reactivity [2]. On the other hand, very small changes in the TCR epitope can have a large impact on the recognition [3], [4]. Due to this complexity, it is difficult to predict the existence, or extent, of cross-recognition by a TCR with a certain specificity for different antigenic peptides. The benefits of successful predictions would be manifold, both in the field of general molecular recognition principles and in the field of immunology or immunotherapy. Indeed, they would allow for a deeper understanding of the shaping of the T cell repertoire during thymic maturation and its regulation in the periphery. Indirectly, this information would provide the key elements for peptide mimetic design, such as optimal rational design of peptidic vaccines in anti-tumor therapy.

Experimental studies of cross-recognition, involving the analysis of the recognition by CTL clones (of known or unknown specificity) of synthetic combinatorial peptide libraries in positional scanning format (PS-SCL), have recently been reported [5]–[8]. The generated data together with the assumption of independent contributions of individual side chains to antigen recognition allowed for a quantitative biometric analysis [9]. In this analysis, self- or pathogen-derived peptide sequences available in public databases were scored and ranked according to their potential reactivity and experimental recognition assays confirmed the cross-reactivity [6]. Although this kind of approach provides an important insight into the peptide sequence diversity in cross-recognition, it does not give any information about the underlying molecular recognition principles. Such information is however crucial for efficient peptide mimetic design.

In this study, we generate three-dimensional (3D) quantitative structure-activity relationships (QSARs) to investigate the 3D criteria for cross-recognition by Melan-A-specific CTLs of diverse peptide sequences bound to the human leukocyte antigen HLA A2 molecule.

The complex relationships between different properties of molecules and their physicochemical or biological activities have commonly been analyzed using neural networks (NN) that are powerful in modelling non-linear relationships [10]. Moreover, genetic algorithms (GA) can be used to select properties that are determinant for those relationships. The usefulness of such so called genetic neural networks (GNN) to generate QSARs from both conventional two-dimensional (2D) descriptors [11], [12], and molecular similarity matrices (SMs) calculated from 3D molecular fields [13], [14] has been reported.

As opposed to conventional 2D descriptors, molecular SMs represent global measures of the resemblance between a pair of molecules based on certain properties, such as shape or electrostatic potential, calculated on a 3D grid. An advantage in using molecular similarity is its efficiency in reducing the raw data dimensionality: the large raw data matrix resulting from 3D grid calculations is compressed into a *cN x N* SM, where *N* is the number of compounds and *c* is a small integer. Nevertheless, when such a QSAR model is used to help the design of new compounds, the use of similarity measures between molecules implies ideally that the optimal biological result has already been achieved by one of the training set structures, and that the design goal is to generate other structures that are almost as good. In this particular study, however, the goal is rather to predict the cross-recognition of peptide-HLA by Melan-A-specific CTLs. Our training set contains both molecules that are totally non cross-recognized on the one hand, and highly cross-recognized on the other hand, which allows a large range of applicability.

In our approach, we first predict the structures of a set of cross-reactive and non-cross-reactive peptides in the HLA A2 molecule using a previously validated *in silico* approach [15]. After computing electrostatic energy and shape data on a peptide-centric grid, we generate a molecular SM. We use the molecular SM and a GNN method proposed by So *et al.* [14] with a 4-1-1 scaled conjugate gradient NN containing seven adjustable parameters to generate 3D-QSAR models that are validated using several different approaches. Initially, we use a partitioned training/test set to test the 4-1-1 NN both for its ability to produce good 3D-QSAR models, characterized by high cross-validated correlation coefficients (*q* ^2^), and its ability to accurately predict experimental cross-reactivities for an external test set. Thereafter, the external test set is included in the training set to produce the final 3D-QSAR models generated for the entire data set. Moreover, the physical validity of all obtained models is analyzed in detail by performing a functional dependence analysis of the individual descriptors. Finally, the robustness of the models obtained from the entire data set is confirmed using y-randomization that involves identical repetitions of the calculation procedure using randomized biological activities; no model with better *q* ^2^ and *r* value could be found with the randomized activities.

In rational peptide modifications for optimization of peptidic anti-tumor vaccines, there is often a need to substitute one or a few side chains that improve MHC affinity without modifying the recognition by the specific CTLs. In the parental Melan-A_26–35_A27L (ELAGIGILTV) peptide, the Ala side chain at position 3 (P3), which is located in front of the hydrophobic D-pocket [16] of HLA A2, is a so-called secondary anchor residue. Substitutions at such secondary positions are delicate and possible conformational changes in the peptide leading to T cell repertoire shifts must be investigated. However, the prediction of such functional modifications based on amino acid sequence information only is impossible. Therefore, we test if our 3D-QSAR models would be able to discriminate between peptides with only one modified side chain and thereby guide the design of closely related analog peptides, despite the large divergence in peptide sequences used to build the QSARs.

To this end, we theoretically predict the structures of all P3-substituted analogs (referred to as ELX) of the parental Melan-A_26–35_A27L peptide bound to HLA A2. After calculations on a grid, the similarity is calculated between each ELX-HLA A2 complex and the different 3D-QSAR descriptor complexes and for each ELX-HLA A2 complex, the cross-reactivity is predicted using the three best 3D-QSAR models obtained previously. Importantly, the predicted cross-recognitions are confirmed experimentally in standard ^51^Cr release assays using six different Melan-A-specific CTL clones.

Our results suggest that despite the complexity of cross-recognition, the properties of the unbound epitope are sufficient to capture most of the information needed, and that the use of 3D-QSARs with high predictive ability opens the door to rational peptide mimetic design.

## Materials and Methods

### 1. Peptide data sets

#### 1.1. Peptide selection for 3D-QSAR model generation

To generate 3D-QSAR models, we use a set of peptides identified in a recent experimental study based on positional scanning synthetic combinatorial peptide libraries (PS-SCLs). In the study, PS-SCLs containing C terminus amidated decapeptides were screened with the Melan-A-specific CTL clone LAU 203/1.5 in functional chromium-release assays [5]. The data was used to generate a scoring matrix for the identification of potentially cross-reactive peptide sequences of self and pathogen origin from the GenPept protein database [6]. The cross-recognition of the retrieved peptides by a set of 17 Melan-A-specific CTL clones was investigated and recorded by assigning +1 for a specific lysis >10%, +2 for >20%, +3 for >40%, +4 for >60% and −1 for lack of significant specific lysis [6]. The recognition was measured in single dose assays with a peptide concentration of 1 μg/ml that in general corresponds to saturating conditions, i.e. the measured recognition contains no contribution from the peptide-HLA A2 affinity.

Importantly, it has been shown that Melan-A-specific CTLs normally completely cross-recognize the Melan-A_26–35_ peptide (EAAGIGILTV), i.e. peptide 22 in Table 1, and the analog Melan-A_26–35_A27L peptide (ELAGIGILTV) [17]. It should however be noted that in situations of non-saturated concentrations of the peptides, the higher binding affinity of Melan-A_26–35_A27L for HLA A2 results in a more efficient recognition of this peptide [17].

**Table 1:** Selected peptides.

Peptide^a^	Sequence	Score^b^	Species	Protein
**Human**				
10	LLAGIGTVPI	15	H. sapiens	PG transporter
22	EAAGIGILTV	56	H. sapiens	Melan-A/Mart-1
**Viral**				
23	RQAGIAGHTY	3	HSV	Capsid protein p40
25	VIAGIGILAI	39	Pseudorabies virus	Glycoprotein GIII
29	NTTDIGIHVV	13	Canine calicivirus	Capsid protein
30	MIAGIGISLI	16	Variola virus	(XHOI-F, O, H, P, Q) genes
37	RITGICFHFG	6	Puma lentivirus 14	GAG polyprotiein
**Bacterial**				
56	MLSGIGIFFI	11	C. trachomatis	Arginine/ornithine antiporter
58	VLSSIGIFPI	3	S. Coelicolor	Putative secreted protein
60	RVTGIGLLTG	9	Synechococcus sp.	REPA
71	RSAFIGIDPA	15	Rhizobium sp.	Y4FN probable ABC transporter permease
72	LLAGIAIGPW	12	E. coli	K+/H+ antiporter
100	FLPSDFFPSV	−17	Hepatitis B virus	Precore/core peptide
101	KLVALGINAV	17	Hepatitis C virus	Polyprotein
102	LLFNILGGWV	−17	Hepatitis C virus	Polyprotein
103	GLYDGMEHTV	−17	H. sapiens	Mage A10 with 2 mutations
104	VLYRYGSFSV	−17	H. sapiens	Gp-100
105	TLVEVTLGEV	−17	H. sapiens	Mage A2, A3, A6, n
106	LLKYRAREPV	−17	H. sapiens	Mage A1, A2, A3, A6
107	ALVETSYVKV	−17	H. sapiens	Mage A3, A12
108	VLPDVFIRCV	−17	H. sapiens	NA17-A
109	LLFGLALIEV	−17	H. sapiens	Mage C2
110	ALSRKVAELV	−17	H. sapiens	Mage A3, n

The 12 most cross-reactive peptide sequences from the experimental work by Rubio-Godoy et al.^6^ (upper part of table) were selected for structure prediction together with a set of 11 non-cross-recognized HLA A2 binding peptide sequences (lower part of table).

a The numbering of the cross-recognized peptides is issuing from the PS-SCL study^6^.

b The cross-reactivity score was calculated from experimental cross-recognition results^6^, see Material and Methods.

Here we use the experimental cross-recognition results obtained by Rubio-Godoy *et al*. [6] to score and rank their peptides from highest to lowest cross-reactivity: for a given peptide, the score is calculated by summing the results (+1, +2, +3, +4 or −1) from the 17 Melan-A-specific clones. This takes the overall probability of cross-reactivity into account since a lack of cross-recognition by a given clone is penalized by the subtraction of −1 from the score. In its form, the cross-reactivity score is useful in the analysis of the probability of cross-recognition by clones with a given specificity of a peptide. The peptides used in the experimental assays [6] were amidated at the C-terminus which might bias recognition. However, if all or many clones recognize a peptide it can not in all cases be only because of the amidation. Therefore, to ensure that we use peptides that are truly cross-recognized by the clones we select only peptides that are recognized by many (at least 7) clones. The 12 most cross-reactive peptides (see Table 1), with scores ranging from 56 (for the parental Melan-A_26–35_ peptide) to 3, are selected for 3D-QSAR generation.

Additionally, a set of 11 HLA A2 binding peptides known not to be recognized by Melan-A-specific CTLs is selected for 3D-QSAR generation (see Table 1). In line with the calculated score above, each of these non-cross-recognized peptides are assigned a score of −17.

#### 1.2. Melan-A26-35A27L P3-substituted analogs: ELX

The Ala residue in peptide position 3 (P3) of the parental Melan-A_26–35_A27L peptide (hereafter referred to ELA) is located in front of the hydrophobic HLA A2 D-pocket [16]. Since secondary pockets are poorly selective [16], the peptide P3 position can be substituted for all natural amino acids without loosing HLA A2 binding, resulting in 19 ELXGIGILTV peptide sequences (referred to as ELX).

### 2. In silico procedures

#### 2.1. Prediction of peptide-HLA A2 structures

The X-ray crystal structure of the ELA peptide in complex with HLA A2 is available from the RCSB Protein Data Bank (http://www.rcsb.org) with the PDB code 1JF118. The structure prediction of each selected peptide (see Sections 2.1.1 and 2.1.2) in the HLA A2 molecule of 1JF1 is carried out as described briefly below and in detail elsewhere [15].

A conformational sampling protocol adapted to the peptide-MHC class I system is used: the sampling is performed using a simple solvation model (ε = 4r) and 1000 simulated annealing (SA) heating-cooling cycles. At the end of each cycle, the conformation of the peptide in the HLA A2 molecule is saved after energy minimization. The complete peptide-HLA A2 complex is present during the entire sampling. The HLA A2 molecule is kept rigid except in two cases (peptides 23 and 72, see Table 1) where the C-terminal side chain (TYR in 23; TRP in 72) is too large to fit into the HLA A2 F-pocket [16]. In these cases, the side chains of Arg97 and Tyr116 are left flexible. To help keeping the N- and C-termini of the peptide in the vicinity of the consensus conformation [16], two NOE distance restraints (±0.4 Å around the X-ray distances in 1JF1) are applied to either end of the peptide. The force constant is set to 5 kcal/(mol Å^2^).

For each of the peptide-HLA A2 complexes, we select the best conformer from the collection of 1000 sampled conformers using an *ad hoc* graph theory clustering approach [15], [18] to cluster the different peptide conformers based on their pairwise heavy atom root mean square deviation (RMSD) values. We rank the clusters based on their conformational free energy (see Equation 1), where the first term is the average effective energy of a cluster (see Equation 2).
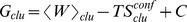
(1)

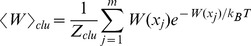
(2)


The effective energy, *W*, which is computed for each conformer, is the sum of the intramolecular energy of the complex and the solvation free energy of the system [19]. The salvation free energy is computed using the Poisson-Boltzmann (PB) continuum model for the solvent [20]. The second contribution to the conformational free energy is the conformational entropy of the cluster (see Equation 3), where the Boltzmann probability and the partition function are evaluated according to Equation 4 and 5.
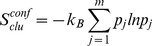
(3)

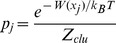
(4)

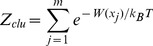
(5)


In the equations, *m* is the number of members in the cluster, *k_B_* is the Boltzmann constant and *T* is the absolute temperature (*T* = 300 K). The term *C* in Equation 1 consists of the free energy contributions from the pure solvent and the ideal contribution from macromolecular translation and rotation; these contributions are independent of conformation and can therefore be considered as a constant that cancels out in the determination of relative free energies [19]. The final structure is chosen as the centre of the cluster with lowest conformational free energy. The centre of a cluster is defined as the conformer having the smallest RMSD sum to all other conformers in the cluster.

The CHARMM [21] (version c31b1) molecular modelling program and the all-atom CHARMM22 protein parameter set [22] are used for all calculations.

#### 2.2. Generation of molecular similarity matrices

Electrostatic and van der Waals interactions are the two key components in any non-covalent ligand-receptor interaction. It has been shown that QSAR predictivity obtained with the van der Waals steric field and the shape steric field are practically equivalent [14]. Since the shape field requires less user variables (i.e. no truncation cut-offs etc.), we generated a double similarity matrix (SM) based on electrostatic energy and binary shape data. For each of the optimally superposed peptide-HLA A2 complexes, the electrostatic energy and the shape data were computed on a peptide-centred grid with 0.5 Å grid spacing. The grid size was designed so as to extend beyond the peptide atomic coordinates of the entire data set by at least 6 Å. Similar conditions were used as in So *et al*. [14], except that they used a grid spacing of 2 Å for calculation of electrostatic energy. The same grid was used for all complexes.

The electrostatic energy was computed with a distance-dependent dielectric constant (ε = 4r) and using a probe with a positive unit charge. To avoid singularities for the electrostatic energy at grid points near the atomic positions, we set the electrostatic energy of points within the van der Waals surface of the molecule to zero. Based on the electrostatic energy distribution, where 90.8% of the values are between +5 kcal/mol and −5 kcal/mol, we truncated electrostatic energies beyond ±5 kcal/mol.

The Hodgkin index [23],
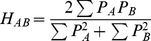
(6)was used to calculate the electrostatic SM. The sum is over all grid points and *P_A_* and *P_B_* indicate the property of interest for molecule *A* and *B*, respectively.

The shape data was computed using a binary function that describes whether a grid point is inside or outside the van der Waals surface of the molecule.

The Carbό index [24],
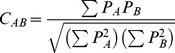
(7)was used to calculate the shape SM. Note that for binary (0 or 1) functions, the Carbό index reduces to the Meyer index [25],
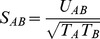
(8)that has been used for shape comparisons. In the Meyer index, UAB is the number of grid points that are inside the common volume of the two molecules, and TA and TB are the number of grid points inside the individual molecular volumes.

The obtained similarity values were in the range of 0.96–1.0 for shape and 0.74–1.0 for electrostatic energy. The lack of lower similarity values is not a limitation since only the variation of the values is important. As will be clear from the results, the similarity variation is sufficient to discriminate well between different peptides.

#### 2.3. Genetic neural network

The genetic neural network (GNN) method proposed by So *et al.* [14] was used to obtain QSARs from a double (shape and electrostatic) molecular SM. In this approach, a genetic algorithm (GA) is used to select molecular descriptors and a neural network (NN) generates a non-linear relationship between these molecular descriptors and the biological activity score of the training set molecules (See Table 1, and paragraph 2.1.1). For the GA we used 250 individuals and 75 evolutionary cycles to assure convergence. We used a 4-1-1 scaled conjugate gradient NN containing 5 adjustable weights and 2 adjustable threshold parameters. The leave-one-out (LOO) cross-validation was performed at each cycle and the cross-validated correlation coefficient
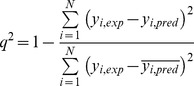
(9)was used as the fitness criterion [13]. The *y_i,exp_* term is the experimental activity and *y_i,pred_* is the predicted activity from the LOO cross-validation. For the maximum possible correlation of the data, *q^2^* equals 1. A value of zero indicates that the predictions are no better than those made randomly.

Since both the GA and the training of the NN are stochastic, we performed 10 (training/test data set) or 50 (entire data set) different GNN runs, each with a different seed for the random number generator. A typical GNN calculation for the entire peptide-HLA A2 data set (23 complexes) required about 1 central processing unit (CPU) hour on an Athlon 64 dual core 4200+.

#### 2.4. Data set partitioning into training and test set

The 7 adjustable parameters used in the 4-1-1 neural network allow for a reasonable division of the entire data set (23 complexes) into a training set of 17 complexes and an external test set of 6 complexes. Because of the small size of the entire data set, a larger external test set could lead to over-fitting of the models obtained using the training set. The partitioning agrees with the guidelines issued by Golbraikh *et al.* [26], according to which the external test set should include at least 5 complexes. Moreover, using a test set of 6 complexes allows us to select 3 cross-reactive and 3 non-cross-reactive complexes each.

The partitioning of the data set was based on ranking of the cross-reactivity scores [26]. First, the complexes were sorted by cross-reactivity and divided into three groups where the four most cross-reactive complexes made up the first group, the four next most cross-reactive complexes made up the second group etc. Second, the three most active complexes of each group were included in the training set and the remaining complexes in the test set. The lack of ranking for the non-cross-reactive complexes (all have score –17) obliged us to randomly pick 3 complexes for the test set. The resulting external test set contained peptides 10, 56,58, 100, 104 and 108 (see Table 1).

### 3. Experimental procedures

#### 3.1. Peptide-HLA A2 multimers

Peptide-HLA multimers are complexes of refolded peptide-HLA/β2-microglobulin trimeric complexes. Complexes were synthesized as earlier described [27], [28]. Briefly, purified HLA A2 heavy chain and β2-microglobulin were synthesized by means of a prokaryotic expression system. The heavy chain was modified by addition of a peptide sequence containing the BirA enzymatic biotinylation site. Heavy chain, β2-microglobulin, and peptide were refolded. The refolded product was biotinylated and conjugated to Streptavidin-phycoerythrin (-PE).

#### 3.2. Cell lines and CTL clones

TAP-deficient T2 cells are HLA A2 human lymphoid cells that are defective in antigen processing, but effectively present exogenously supplied peptides [29].

Peripheral blood mononuclear cells (PBMC) were isolated by Ficoll-Hypaque (Beckman-Coulter, Fullerton, CA) either from a healthy HLA A2 blood donor (BC25) or from an HLA A2 metastatic melanoma patient (LAU 203, described elsewhere [30]). ELA-HLA A2 multimer^+^ CD8^+^ T lymphocytes were purified from PBMC by flow cytometry cell sorting and were cloned by limiting dilution culture in the presence of PHA, allogenic irradiated PBMC and human recombinant IL-2, as previously described [31]. T lymphocyte clones were maintained in complete culture medium (RPMI medium supplemented with 10% human serum, amino acids, antibiotics) in the presence of hrIL-2 at 150 IU/ml. Clones 25-R3 and 25-R35 are two Melan-A-specific CTL clones derived from the healthy donor BC25. Clones 203-R1, 203-R2, 203-R3 and 203-R7 are four Melan-A-specific CTL clones derived from the patient LAU 203. The Influenza Matrix peptide (FluMa_58–66_) specific T cell clone was obtained from a healthy donor by limiting dilution.

Written informed consent was obtained from all patients or healthy individuals involved in this study. The study was approved by the ethical committee of the Medical Faculty, University of Lausanne, and the Ludwig Institute for Cancer Research.

#### 3.3. Chromium release assay for ELX recognition

After labelling with ^51^Cr during 1h at 37°C followed by extensive washing, target cells (T2 cells) were incubated with effector cells (T lymphocytes) at an E/T ratio of 10/1 during 4h at 37°C in V-bottomed microtiter plates in the presence of serial dilutions of the indicated synthetic peptide. Chromium release was measured using LumaPlate-96 plates (PerkinElmer, Wellesley, MA) and a TopCount-counter (PerkinElmer). The six different Melan-A-specific CTL clones described in Section 2.3.2 were used for the assay. Two independent experiments were performed for each clone.

The absolute functional avidity of a CTL clone for a specific peptide-HLA A2 complex was defined as the peptide concentration (in Molar) required to induce 50% of the maximal lysis capacity (EC50) of the clone. To determine the absolute functional avidity from the raw data set, a regression analysis of the linear domain of the titration curve was performed. For comparison, the logarithm of the relative functional avidity of a given ELX analog to ELA was calculated: log10(EC50*_ELA_*/EC50*_ELX_*). The average over the independent experiments was calculated.

#### 3.4. ELX-HLA A2 competition assay

Various concentrations of the competitor peptides (50*μ*l) were incubated with ^51^Cr-pulsed T2 cells (50*μ*l; 1000 cells/well) for 15 min at room temperature. The antigenic Influenza Matrix peptide, FluMa_58–66_, was added at a concentration of 0.1 nM (50*μ*l) together with a FluMa_58–66_-specific CTL clone (50* μ*l; 5000 cells/well). Chromium release was measured after 4 h incubation at 37°C in a TopCount NXT*^TM^* (Packard) plate reader. The normalized percent specific lysis was calculated as follows: 100x(percent specific lysis with competitor)/(percent lysis with FluMa_58–66_ (at 0.1 nM)).

## Results and Discussion

### 1. Prediction of peptide-HLA A2 structures for 3D-QSAR model generation

**Figure 1:**
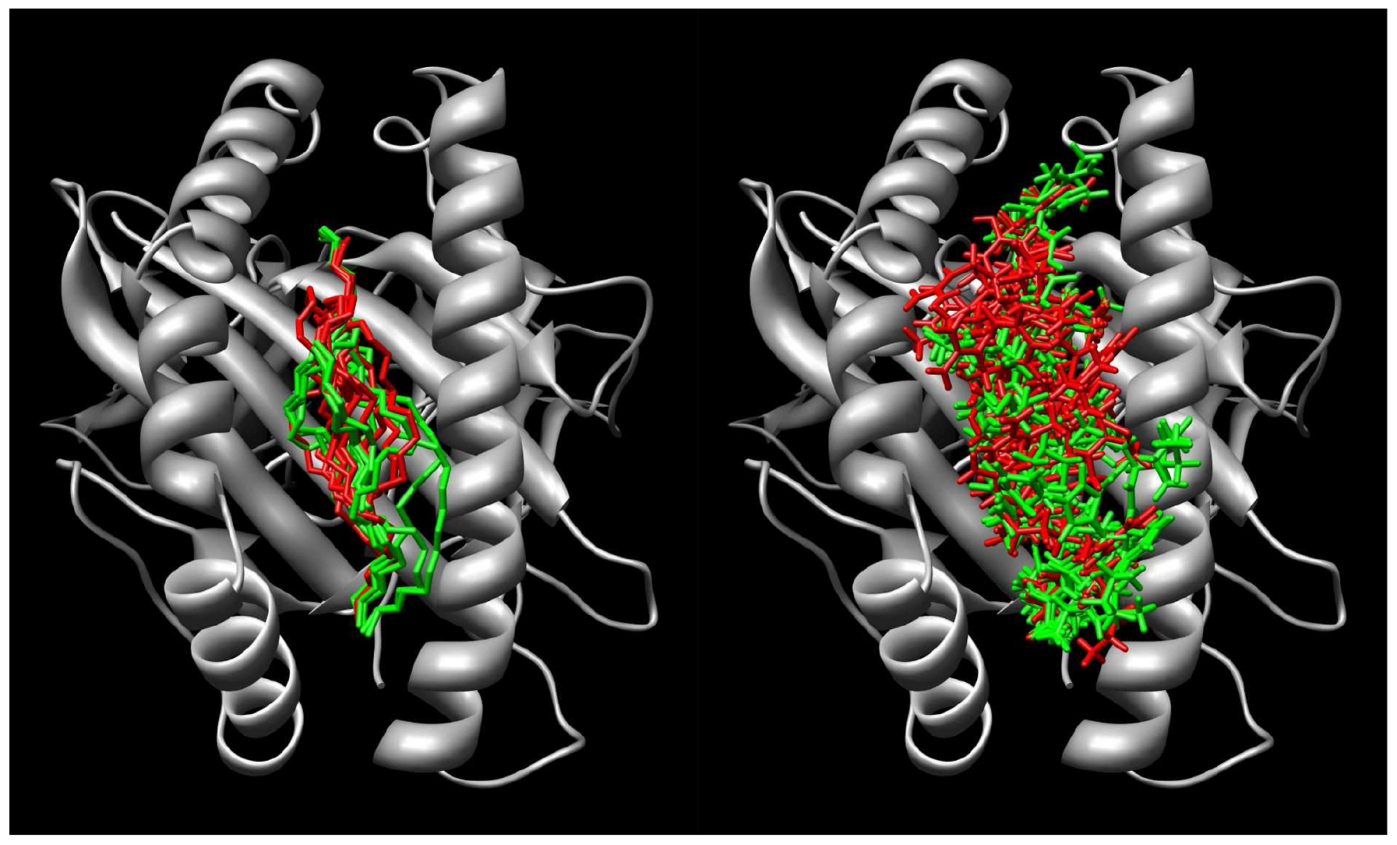
The predicted structures of the 23 selected peptides are visualized in the HLA A2 molecule (gray). The backbone (left image) and all atoms (right image) are visualized for the cross-reactive (green) and noncross-reactive (red) peptides. The N-termini of the peptides are in the upper part of the image. The image was generated using the Chimera program [33].

**Figure 2:**
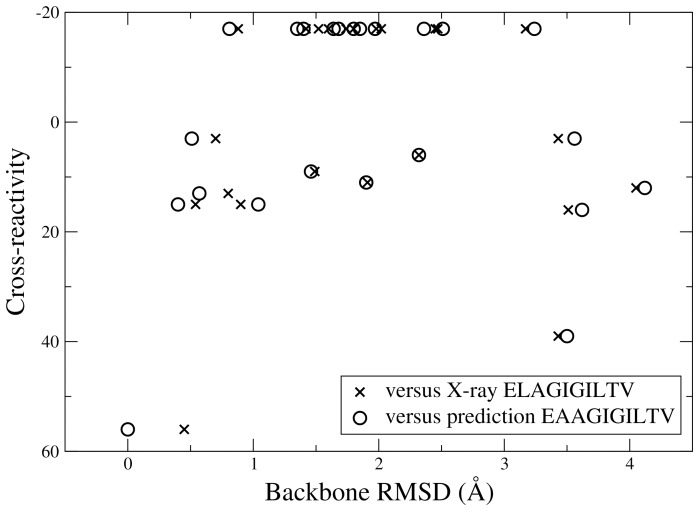
The backbone RMSD to the X-ray structure of the parental peptide Melan-A_26–35_A27L (ELAGIGILTV) or to the predicted structure of the parental peptide Melan-A_26–35_ (peptide 22; EAAGIGILTV) versus the experimental cross-reactivity: no correlation can be observed.

The conformations of the 23 selected peptides (see Table 1) in their fixed HLA A2 environment were predicted using a previously described *ab initio* approach [15]. For a brief description of the approach, see Material and Methods. The predicted structure of Melan-A_26–35_ (peptide 22 in Table 1; EAAGIGILTV), is very similar to the X-ray crystal structure of the Melan-A_26–35_A27L (ELAGIGILTV) peptide: backbone RMSD is 0.45 Å and heavy atom RMSD (including C*β* of the side chain in position 2) is 1.24 Å. The predicted structures of the remaining peptides cover a wide range of different conformations (see Figure 1) and, as shown in Figure 2, there is no correlation between the backbone RMSD to either ELAGIGILTV or EAAGIGILTV and the experimental cross-reactivity (correlation coefficients r  = −0.05 and −0.08). Hence, for this set of peptides with highly diverse sequences, there is no trivial way of predicting cross-recognition by Melan-A-specific CTLs of a peptide in HLA A2 considering only its backbone similarities to the parental peptides (Melan-A_26–35_ or Melan-A_26–35_A27L).

### 2. 3D-QSAR model generation and validation using training/test data set

Initially, we tested the 4-1-1 neural network for both its ability to produce good 3D-QSAR models (characterized by a high *q* ^2^) and its ability to accurately predict experimental cross-reactivities of peptide-HLA A2 complexes for an external test set, i.e. complexes that were not used for the model development. The entire data set (23 complexes) was divided into a training set of 17 complexes and an external test set of 6 complexes, see Material and Methods. No information from the external test set was used for the model development.

**Figure 3:**
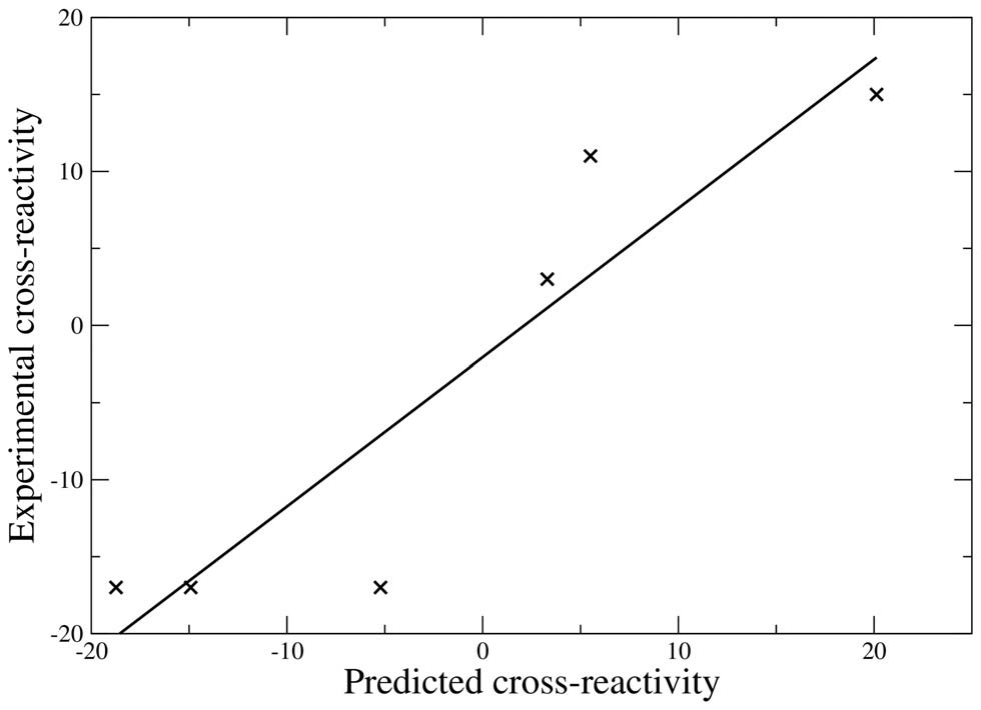
Correlation between the predicted and experimental cross-reactivities for the external test set (6 complexes). The correlation coefficient is 0.92; the slope is 0.97 and the intercept is −2.1.

The best 3D-QSAR model is characterized by a good cross-validated correlation coefficient (*q* ^2^ = 0.75) for the training set, indicating that the necessary, but not sufficient [32], condition for a good 3D-QSAR model is fulfilled. To evaluate the real predictivity of the model, the correlation coefficient (*r*) between the predicted and the experimental cross-reactivities is computed for the external test set. Indeed, the high predictive ability of the model is confirmed with a *r* value of 0.92 (see Figure 3). Moreover, a slope close to 1 (0.97) and an intercept close to 0 (−2.1) is obtained for the regression line, indicating that the model is close to the *ideal model* defined by Golbraikh *et al.* [32]. Additionally, the high *r* value for the external test set shows that the 4-1-1 neural network with 7 adjustable parameters is not over-fitted for the reduced training set (17 complexes).

**Figure 4:**
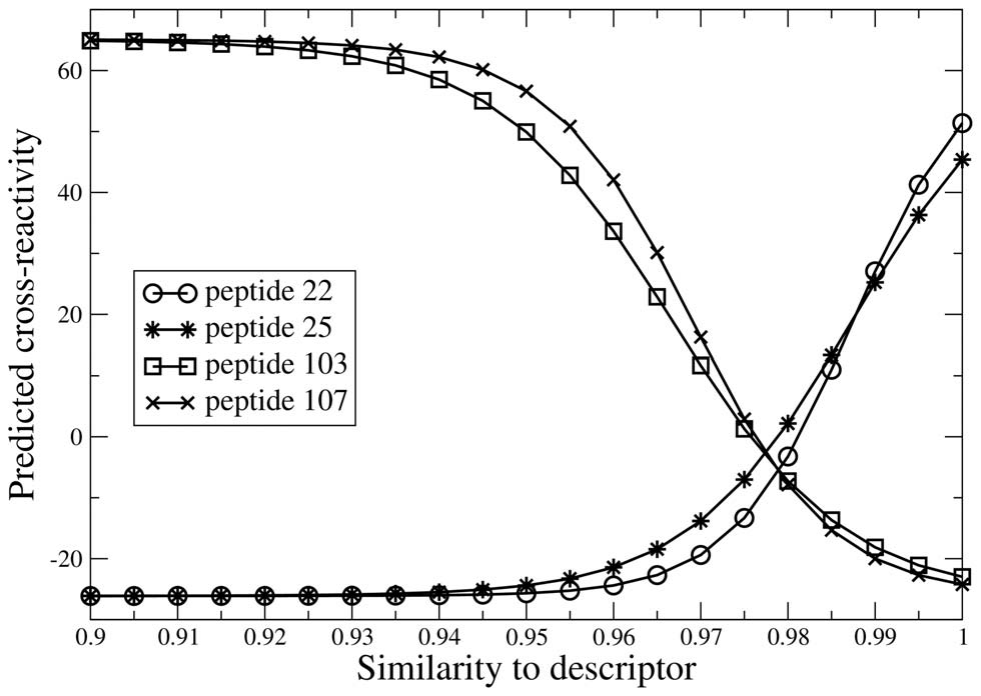
Predicted cross-reactivity as a function of the similarity to the four different descriptors in the model obtained for the reduced training set (17 molecules). It can be observed that an increased similarity to the cross-reactive peptides 22 and 25 increases the predicted cross-reactivity, while an increase in similarity to one of the non-cross-reactive peptides 103 and 107 decreases the predicted cross-reactivity.

The physical relevance of the best 3D-QSAR model, which uses the shape similarity to peptides 22, 25, 103 and 107 to predict the cross-reactivity, was investigated by performing a functional dependence analysis of the individual descriptors. Using the model, a plot was generated for each similarity descriptor by scanning the corresponding similarity value while keeping all other descriptors fixed at a value equal to the average similarity observed in the set (see Figure 4). An increased similarity to the cross-reactive peptides 22 and 25 increases the predicted cross-reactivity, while an increased similarity to one of the non-cross-reactive peptides 103 and 107 decreases the predicted cross-reactivity. This supports the validity of the model.

### 3. 3D-QSAR model generation and validation using the entire data set

With the aim to produce the best possible 3D-QSAR models, the external test set was included in the training set and 3D-QSAR models were generated from the entire data set (23 complexes).

**Figure 5:**
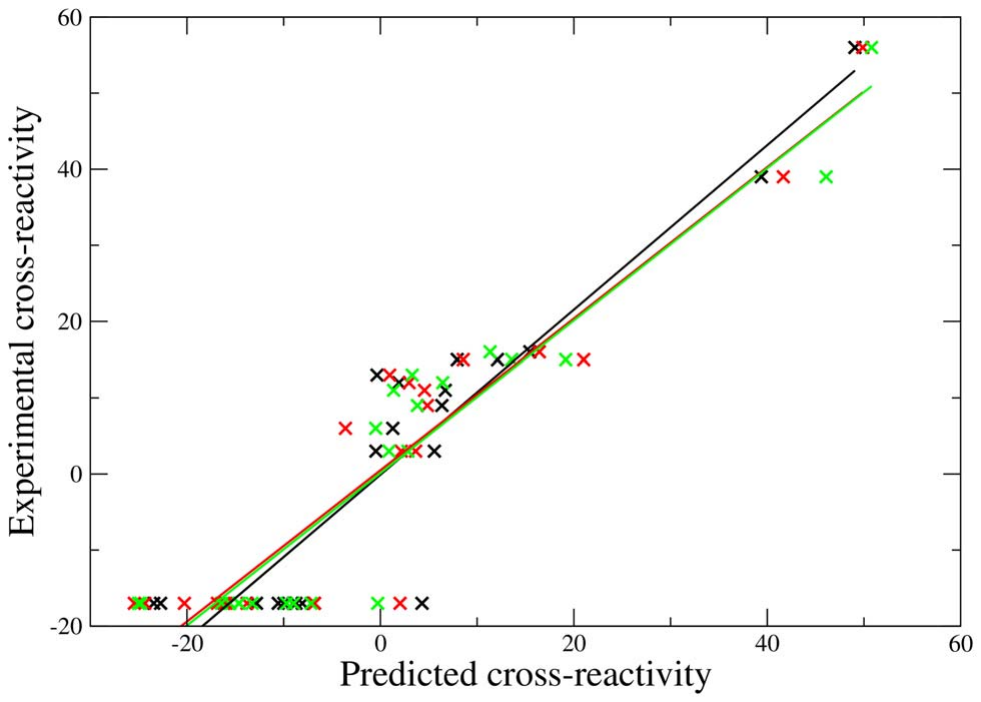
Correlation between the predicted and experimental cross-reactivities for the training set (23 molecules). The three different 3D-QSAR models (black, red, green) give very similar results (*r* = 0.93–0.94).

The three best 3D-QSAR models are from a predicitivity point of view practically indistinguishable, such that one model could not be chosen over the other two. They are characterized by high cross-validated correlation coefficients (*q* ^2^ = 0.78-0.79) and corresponding high correlation coefficients (*r* = 0.93–0.94). The predicted versus the experimental cross-reactivity is plotted together with regression lines in Figure 5. All three models have three similarity descriptors in common: the shape similarity to peptides 22, 25 and 103. The fourth descriptor is the shape similarity to peptide 105, 107 or 110. It is noteworthy that the best model obtained from the partitioned training/test data set (see above) contains the same four descriptors as one of the three models obtained here. The best model containing at least one electrostatic similarity descriptor is characterized by a *q* ^2^ value of 0.73 and an *r* of 0.87; the electrostatic similarity to peptide 22 (Melan-A_26–35_) is one of the descriptors. Only the three best models (see above) will be considered below.

**Figure 6:**
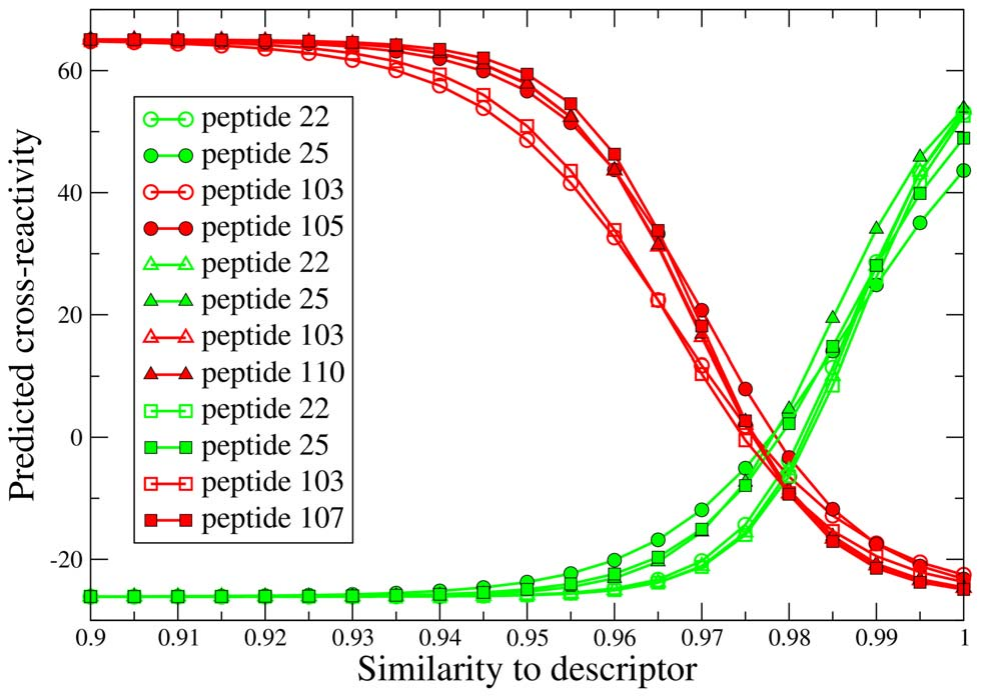
Predicted cross-reactivity as a function of the similarity to the different descriptors in the three models obtained for the entire data set (23 molecules). The three different models are indicated by circles, triangles and squares, respectively. The descriptors are colored according to cross-reactivity: cross-reactive descriptors, i.e. peptides 22 and 25, are colored green and non-cross-reactive descriptors, i.e. peptides 103 and 105/107/110, are colored red. It can be observed that an increased similarity to the cross-reactive peptides increases the predicted cross-reactivity, while an increase in similarity to one of the non-cross-reactive peptides decreases the predicted cross-reactivity.

As for the model obtained from the training/test data set, the physical relevance of the models was investigated by performing a functional dependence analysis of the individual descriptors. As shown in Figure 6, the results are similar to those obtained for the training/test data set. An increased similarity to the cross-reactive peptides (22, 25) increases the predicted cross-reactivity, while an increase in similarity to one of the non-cross-reactive peptides (103 and 105/107/110) decreases the predicted cross-reactivity. Again, the results support the physical validity of the model.

**Figure 7:**
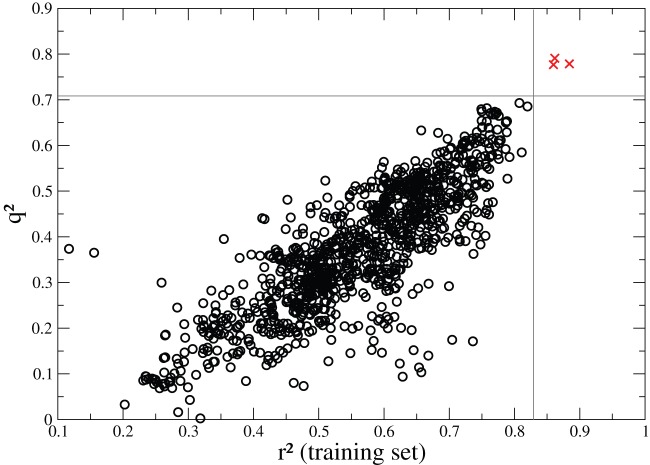
Scatter plot for *q* ^2^ against *r* ^2^ (calculated on the training set) for the three real 3D-QSAR models (red crosses) and for those obtained with randomized cross-reactivities (black circles). The real 3D-QSAR models are well-separated from the random cases, implying robust real models.

As a final model validation, the robustness of the models was evaluated using a so-called y-randomization: the GNN calculation procedure was repeated with randomly shuffled cross-reactivities. If some QSARs with high *q* ^2^ values were still obtained using randomized activities, the significance of the real QSARs (non-randomized activities) would be suspect. A hundred different randomizations of the cross-reactivities were performed and the 10 best models obtained for each randomization are plotted together with the three best real 3D-QSARs in Figure 7. Noticeably, the nature of the Y vector, which contained the same value for all the non-cross recognized peptides, decreased the scrambling effect of the Y-randomization procedure. This biased the Y-randomized models toward larger q^2^ values compared to QSAR models treating more conventional biological data. In addition, for each of the 100 Y-randomizations, we have performed 50 GNN runs, and, for the clarity of the figure, we only represented the 10 best models for each on Figure 7 and deleted those with negative q^2^ values. This procedure focused Figure 7 on the largest q^2^ values for the Y-randomized models, which were indeed those leading to the most challenging assessment of our real 3D-QSAR models. Despite this difficult situation, it can be observed that the real 3D-QSAR models are well-separated from the random cases, implying that the real models cannot efficiently account for physically non-relevant data.

To test the sensitivity of the generated models to the values assigned for the cross-reactivities, the peptides were also scored and ranked according to the *number of clones* that experimentally recognized the peptide-HLA A2 complexes [6]. The score ranged from 17 for the most cross-reactive peptide (peptide 22) to 0 for the non-cross-reactive peptides. 3D-QSAR models were generated with the 4-1-1 GNN approach: the three best models were characterized by similar cross-validated correlation coefficient values (*q* ^2^ = 0.76–0.77) as before. Values similar to before were also obtained for the correlation coefficient (*r* = 0.91–0.93) for the training set. Importantly, the same four descriptors as before were selected for the models: the shape similarity to peptides 22, 25, 103 and 107/110. These results show that the obtained 3D-QSAR models were robust with respect to the type of scoring that were used to describe the cross-reactivity. On the other hand, a binary score, i.e. 0 for non-cross-reactive and 1 for cross-reactive, proved to be too simplistic. Here, high *q* ^2^ and *r* values were obtained for models with randomized cross-reactivities.

### 4. Interpretation of the 3D-QSAR models

Molecular similarity indices are a very different kind of descriptors from conventional 2D descriptors: they represent a global measure of the resemblance between a pair of molecules based on certain attributes, such as shape or electrostatic energy. Therefore, although the approach described here produces reliable 3D-QSAR models, the global character of the descriptors is more difficult to interpret than conventional 2D descriptors. Nevertheless, the 3D-QSAR models obtained in this study suggest that *shape* similarities/differences between a given peptide-HLA A2 complex and the descriptor complexes are sufficient for a correct prediction of the cross-reactivity of the former.

It is noteworthy that models containing *electrostatic* similarity descriptors were generated with high *q* ^2^ values (0.71–0.73). Interestingly, a descriptor in common in these models is the molecular electrostatic similarity to peptide 22, i.e. the parental Melan-A_26–35_ peptide. In fact, for all 3D-QSAR models generated from the entire data set, peptide 22 was selected as either a shape or an electrostatic similarity descriptor. The omnipresence of the Melan-A_26–35_ peptide as a similarity descriptor to predict cross-recognition by Melan-A-specific CTLs is expected since QSAR models, in order to be highly predictive, may need to select a descriptor corresponding to a highly active compound.

The lack of electrostatic descriptors in the three best 3D-QSAR models obtained in this study is likely due to the overall non-polar character of the parental Melan-A_26–35_ (EAAGIGILTV) and the overall non-polar character observed in most cross-reactive peptide amino acid sequences (see Table 1). It is, however, less expected to find that the descriptors based on the similarity to non-cross-reactive peptides are related to shape, even though these sequences in general contain a significant number of polar or charged side chains. Moreover, based on previously published X-ray crystallographic structures, hydrogen bonds are known to be important in the interaction between TCR and peptide-MHC, and an addition of 2D descriptors mapping such putative contacts could be useful to generate QSAR models with high predictive power.

No simple linear relationship could be observed between the shape similarity to *individual* descriptors and the experimental activity (data not shown). Hence, the high predictivity of the 3D-QSAR models suggests that the generated non-linear relationship between *several* shape similarity descriptors and cross-reactivities is crucial for the successful outcome.

Together, these results suggests that the 3D-QSAR models are very efficient, and that a non-linear relationship is indeed necessary for successful prediction of the probability of cross-recognition by Melan-A-specific CTLs of peptides with diverse sequences. Finally, although the absence of TCR influence in the structure predictions may be a source of error in the prediction of cross-reactivity based on those structures, the results suggest that properties of the unbound epitope are sufficient to capture most of the information to determine the cross-reactivity.

### 5. Application of the 3D-QSAR models to rational peptide modifications: an additional external test set

Above we show that the 3D-QSAR models are successful in discriminating between cross-reactive and non-cross-reactive peptides with diverse sequences. Here, we test if these 3D-QSAR models are able to discriminate between peptides with only one modified side chain and thereby guide the design of closely related analog peptides, despite the large divergence in peptide sequences used to build the QSARs.

To this end, we theoretically predict the structures of all P3-substituted analogs of the parental Melan-A_26–35_A27L peptide bound to HLA A2, referred to as ELX-HLA A2, see Material and Methods. After shape data calculation on the same grid as before, the similarity is calculated between each ELX-HLA A2 complex and the different 3D-QSAR descriptor complexes, i.e. peptide 22, 25, 103 and 105/107/110. For each ELX peptide, its cross-reactivity is predicted using each of the three 3D-QSAR models. According to a consensus scoring approach [11], the final predicted cross-reactivity for each analog peptide is calculated as the average of the results from the three models. Although spanning a wide range, i.e. from 27 (for ELS) to −12 (for ELW), the values are within the range of the experimental cross-reactivities, see Table 1. Interestingly, the score for ELS indicates that it should be very highly cross-recognized by Melan-A-specific CTLs. On the contrary, ELY (score = −8), ELK (-10) and ELW (-12) should not be cross-recognized. Most other ELX peptides, like the second (ELC: 20) and third (ELG: 18) best scored peptides, should be expected to be well recognized although to a lesser degree than the ELS peptide. Interestingly, ELT (0) belongs to these peptides. Hence, although this analog peptide contains threonine that has similar physico-chemical properties as serine, except for the larger volume due to the additional methyl group, it is predicted to be less cross-recognized than ELS (27).

To test if these theoretical results can be confirmed experimentally, we evaluated the cross-recognition by six different Melan-A-specific CTL clones in standard ^51^Cr release assays, see Material and Methods. The experimental relative cross-recognition of ELS and ELT peptides versus the parental peptide by the six different Melan-A-specific CTL clones is given in Table 2. It can be observed that the ELS analog is more frequently cross-recognized than the ELT analog: whereas 5 of 6 Melan-A-specific CTLs recognize the ELS analog within 1.5 log of the parental peptide, only 3 of 6 CTLs recognize the ELT analog. Moreover, the average cross-recognition relative to ELA over all six CTL clones is −0.75 for ELS and −0.97 for ELT, again showing the higher cross-reactivity of ELS compared to ELT.

**Table 2:** Cross-recognition results for six different Melan-A-specic CTL clones in a chromium release assay: the logarithm of the relative functional avidity of the ELX analogs to ELA is given.

Seq^a^	Melan-A-specic CTL clones											
	203-R7		25-R3		203-R2^b^		203-R1		203-R3		25-R35	
ELA	0.00^c^	0.00^d^	0.00	0.00	0.00	0.00	0.00	0.00	0.00	0.00	0.00	0.00
ELS	−0.59	0.14	−0.41	0.13	−0.07	0.00	−0.01	0.10	−0.99	0.01	−2.44	0.50
ELT	−0.19	0.32	−2.17	0.11	0.09	0.00	−0.05	0.15	−1.88	0.08	−1.59	0.62

a The P1-P3 peptide sequence of the ELX analog.

b Only one experiment was done with the 203-R2 clone.

c The logarithm of the relative functional avidity of the ELX analogs compared to ELA was calculated as log10(EC50_ELA_/EC50_ELX_). A value of −1.00 means that the molar concentration of the ELX peptide needs to be 10 times higher than ELA to achieve the same activity, i.e. 50% of maximal lysis. The average over the independent experiments for each CTL clone is given.

d Standard deviation. By the denition of the score, the standard deviation for ELA is zero.

**Figure 8:**
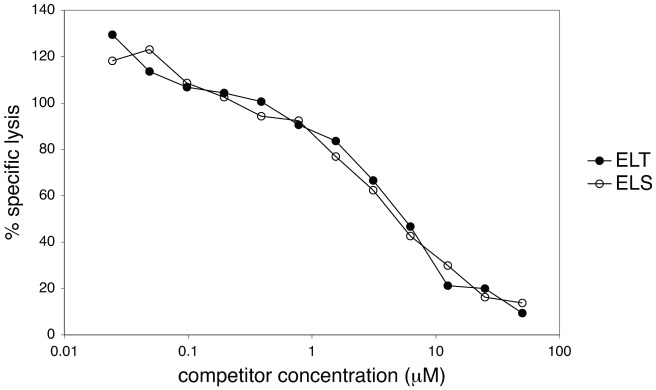
The binding affinity of ELS and ELT for HLA A2 was evaluated in competition assays. (See Material and Methods.) The average of two independent, but very similar, results is shown: the two analog peptides competed with very similar efficiency indicating that their affinities for HLA A2 were practically indistinguishable. Note that values are relative to specific lysis with the Influenza Matrix peptide FluMa_58–66_ by the FluMa_58–66_ specific clone and can therefore take values >100%, see Material and Methods.

In these assays, saturating conditions are not satisfied and potential differences in ELX-HLA A2 binding affinity might contribute to the cross-recognition results. In order to exclude that the cross-recognition results obtained for the ELS and ELT analogs were due to differences in affinity for HLA A2, we performed competition assays using either peptide as competitor, see Material and Methods. The competition results from two independent experiments are very similar and the average of normalized specific lysis (%) from the two experiments is plotted in Figure 8. The two analog peptides compete with very similar efficiency indicating that their affinities for HLA A2 are practically indistinguishable and that the observed differences in cross-recognition (see above) are mainly due to the interaction of the TCRs with peptide-HLA A2.

**Figure 9:**
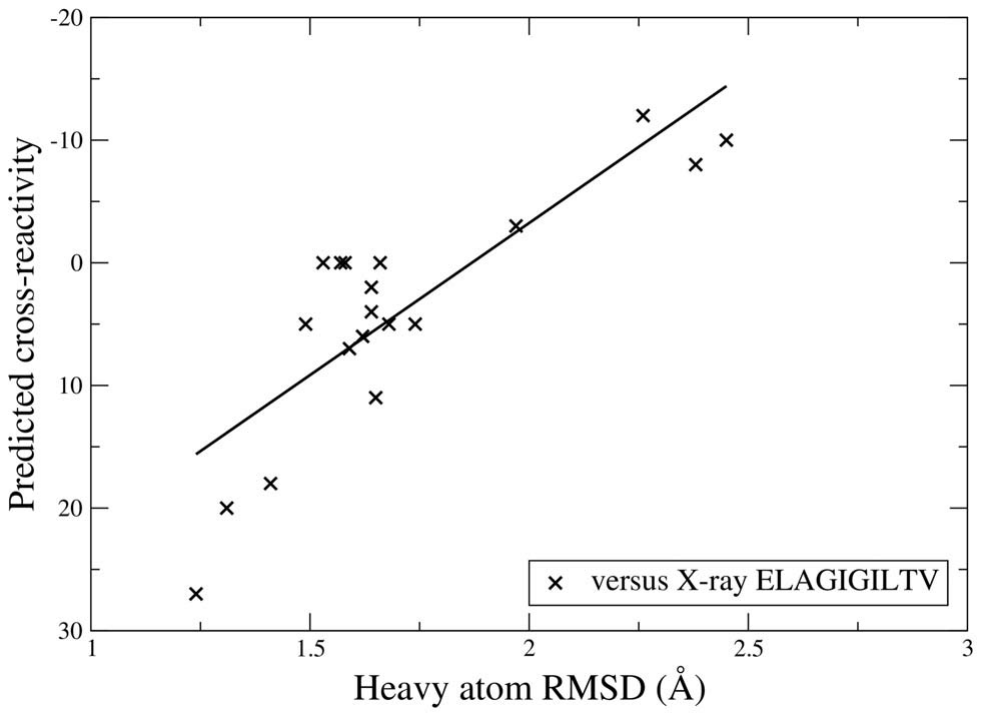
The heavy atom RMSD to the X-ray structure of the parental peptide Melan-A_26–35_A27L (ELAGIGILTV) versus the predicted cross-reactivity for the ELX set: a correlation can be observed (correlation coefficient:−0.84). In fact, an increased RMSD to the parental peptide corresponds to a lower predicted cross-reactivity.

In contrast to the lack of correlation between RMSD and cross-reactivity observed for the diverse data set used for 3D-QSAR model generation (Figure 2), here we observe a correlation between RMSD to the parental ELA peptide and the predicted cross-reactivity of ELX, see Figure 9. In fact, heavy atom RMSD values between the predicted structures of the ELX peptides and the X-ray structure of ELA (excluding side chain atoms beyond Cα at the substitution site) is linearly related to the predicted cross-reactivity of ELX with a correlation coefficient of −0.84. Similar results were obtained using backbone RMSD values (correlation coefficient: −0.89).

Taken together, these results show that for mono-substituted peptides the RMSD to the parental peptide is sufficient for determining whether a peptide will be cross-reactive or not. However, the results also show that the 3D-QSAR models obtained from the data set of diverse peptide sequences (see Table 1) are equally successful in discriminating between peptides with single amino acid substitutions.

## Conclusion

In this study, we have investigated the use of 3D-QSARs in the prediction of the probability of cross-recognition by Melan-A-specific CTLs of peptides with different sequences. We show that the use of 3D molecular descriptors (in the form of a similarity matrix) and a 4-1-1 genetic neural network allow for the generation of robust 3D-QSAR models that are characterized by a high predictive ability as evaluated on both a partitioned training/test set and the entire data set of highly diverse peptide sequences. Moreover, the 3D-QSARs could not be replaced by trivial correlations between structure and cross-reactivity. Application of the 3D-QSARs to an additional external test set of mono-substituted peptides shows that the models are also capable of distinguishing between different degrees of cross-reactivity for these peptides. Importantly, experiments confirm the theoretical results.

Taken together, our results suggest that 3D-QSARs can be highly successful in predicting the probability of cross-recognition by specific CTLs of different peptides. This allows for efficient rational peptide mimetic design.

## Supporting Information

Appendix S1 **HLA-A2-1jf1.pdb.** 3D structure of the HLA-A2 molecule used for the docking. It corresponds to the 1JF1 entry of the PDB.
(PDB)

Appendix S2 **peptides dock4.pdb.** 3D structures for the calculated binding modes of the 23 peptides shown in Figure 1. In PDB format, following the dock4 specifications to make the visualization easier in UCSF Chimera, using the ViewDock plugin. In the right-most column, – 3SG corresponds to the peptides shown in Figure 1. – 1SG corresponds to 2 residues of HLA-A2 (Arg97 and Tyr116) that were considered flexible during the docking of peptides 23 and 72.
(PDB)
